# The role of mitochondrial dynamics and mitophagy in skeletal muscle atrophy: from molecular mechanisms to therapeutic insights

**DOI:** 10.1186/s11658-024-00572-y

**Published:** 2024-04-23

**Authors:** Yuhang Lei, Mailin Gan, Yanhao Qiu, Qiuyang Chen, Xingyu Wang, Tianci Liao, Mengying Zhao, Lei Chen, Shunhua Zhang, Ye Zhao, Lili Niu, Yan Wang, Li Zhu, Linyuan Shen

**Affiliations:** 1 Farm Animal Genetic Resources Exploration and Innovation Key Laboratory of Sichuan Province, Sichuan Agricultural University, Chengdu, 611130 China; 2 Key Laboratory of Livestock and Poultry Multi-Omics, Ministry of Agriculture and Rural Affairs, College of Animal and Technology, Sichuan Agricultural University, Chengdu, 611130 China

**Keywords:** Mitochondrial dynamics, Mitophagy, Skeletal muscle atrophy, Intermodulation, Molecular mechanism, Prevention and treatment

## Abstract

Skeletal muscle is the largest metabolic organ of the human body. Maintaining the best quality control and functional integrity of mitochondria is essential for the health of skeletal muscle. However, mitochondrial dysfunction characterized by mitochondrial dynamic imbalance and mitophagy disruption can lead to varying degrees of muscle atrophy, but the underlying mechanism of action is still unclear. Although mitochondrial dynamics and mitophagy are two different mitochondrial quality control mechanisms, a large amount of evidence has indicated that they are interrelated and mutually regulated. The former maintains the balance of the mitochondrial network, eliminates damaged or aged mitochondria, and enables cells to survive normally. The latter degrades damaged or aged mitochondria through the lysosomal pathway, ensuring cellular functional health and metabolic homeostasis. Skeletal muscle atrophy is considered an urgent global health issue. Understanding and gaining knowledge about muscle atrophy caused by mitochondrial dysfunction, particularly focusing on mitochondrial dynamics and mitochondrial autophagy, can greatly contribute to the prevention and treatment of muscle atrophy. In this review, we critically summarize the recent research progress on mitochondrial dynamics and mitophagy in skeletal muscle atrophy, and expound on the intrinsic molecular mechanism of skeletal muscle atrophy caused by mitochondrial dynamics and mitophagy. Importantly, we emphasize the potential of targeting mitochondrial dynamics and mitophagy as therapeutic strategies for the prevention and treatment of muscle atrophy, including pharmacological treatment and exercise therapy, and summarize effective methods for the treatment of skeletal muscle atrophy.

## Introduction

Skeletal muscle, which accounts for approximately 40% of total body weight, is critical for movement control, postural support, heat production, and metabolic balance [1]. However, the delicate balance between protein synthesis and degradation in skeletal muscle is disrupted when individuals experience various adverse pathological and physiological stressors, resulting in a decrease in muscle mass and strength [2]. This process is commonly referred to as skeletal muscle atrophy and is characterized by a reduced cross-sectional area of muscle fibers, loss of muscle mass and strength, and loss of balance between protein synthesis and degradation. Skeletal muscle atrophy has significant health consequences, and it was recognized as a distinct disease according to its own International Classification of Diseases, 10th Revision, Clinical Modification (ICD-10-CM) code (M62.84) in 2016 [3]. Skeletal muscle atrophy not only is prevalent among older individuals but also may occur secondary to peripheral nerve injury, cancer, diabetes, heart failure, and other diseases. Consequently, skeletal muscle atrophy can be categorized into primary and secondary skeletal muscle atrophy based on the underlying predisposing factors [4]. Muscle fiber type conversion occurs in both types of skeletal muscle atrophy, involving transitions from slow muscle fibers to fast muscle fibers, as well as from fast muscle fibers to slow muscle fibers [5, 6]. The impact of muscle atrophy is not limited to a decrease in quality of life; it is also associated with increased morbidity and mortality [7]. On the other hand, maintaining skeletal muscle health is closely related to reducing morbidity and mortality, which highlights the importance of maintaining the best muscle mass in the human body [8]. Currently, skeletal muscle atrophy has evolved into a critical global clinical issue, necessitating the development of innovative treatment approaches.

Mitochondria are important sites for energy production in the body, and skeletal muscle contraction requires a large amount of energy supply. To sustain normal muscle function and movement, the energy needed for skeletal muscle contraction must align with the ATP molecules produced by mitochondria. Research has demonstrated that skeletal muscle fibers, also known as mitochondrial pools, have two unique mitochondrial populations, subsarcolemmal (SS) mitochondria and intermyofibrillar (IMF) mitochondria [9]. These separate mitochondrial pools work together and generate ample ATP through oxidative phosphorylation (OXPHOS) to support the basic and contractile energy needs of the skeletal muscle network. To elucidate the pathogenesis of skeletal muscle atrophy, various biological mechanisms, including the ubiquitin‒proteasome system, autophagy–lysosomal system, cysteine aspartic acid system, and calpain system, have been proposed [10]. Certainly, there is compelling evidence supporting the idea that the accumulation of mitochondrial dysfunction contributes to the energy deficit and alterations in the balance between protein synthesis and degradation observed in skeletal muscle [11–13]. This dysfunction can promote the development of skeletal muscle atrophy by activating various pathways, including accumulation of reactive oxygen species (ROS), decreased mitochondrial biogenesis, imbalance in mitochondrial dynamics, and impaired mitophagy [14, 15]. These evidences indicate that mitochondria play a particularly prominent role in maintaining skeletal muscle health, emphasizing its possibility and importance in preventing skeletal muscle atrophy.

**Fig.1:**
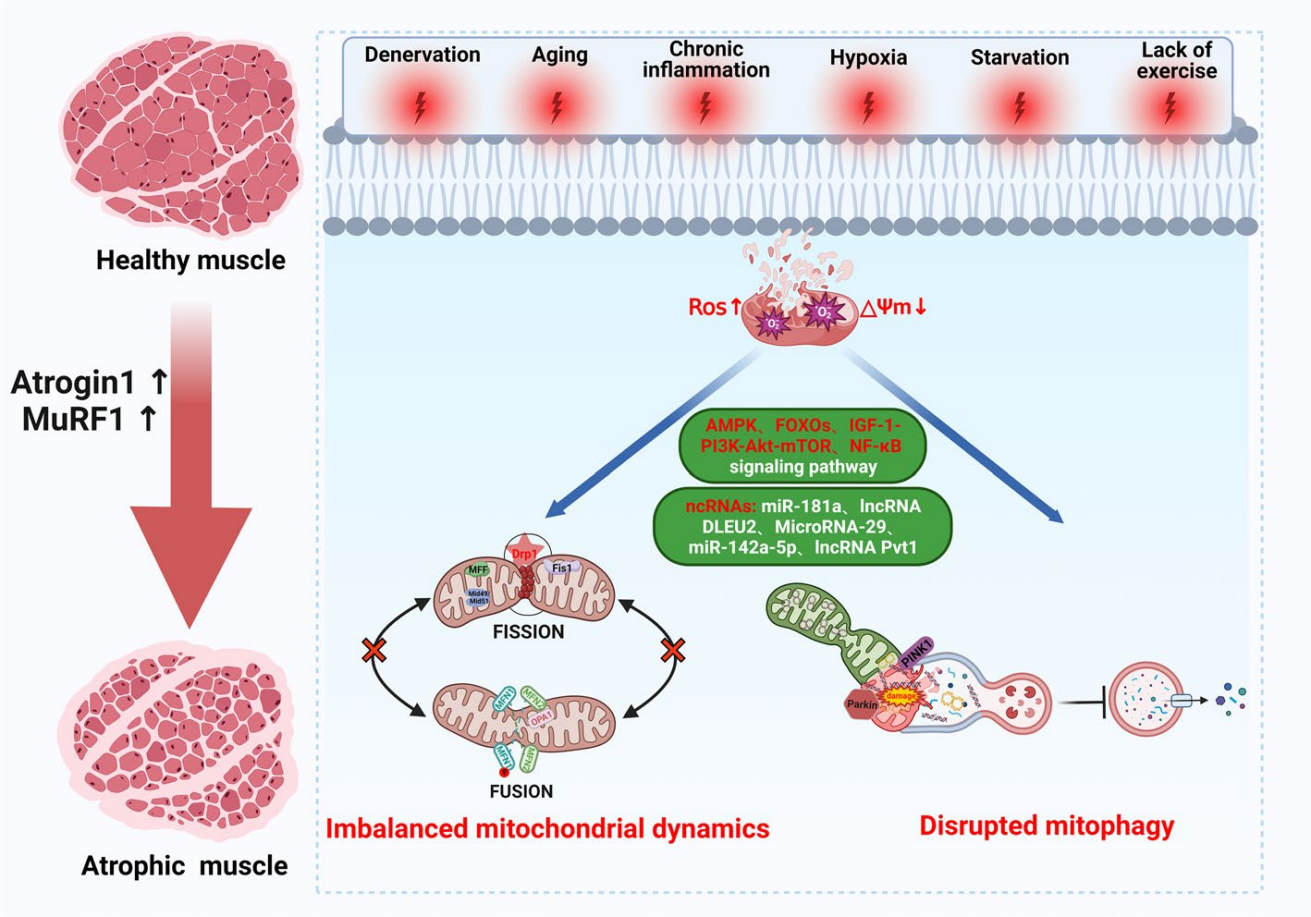
The molecular mechanisms of skeletal muscle atrophy associated with mitochondrial dynamics and mitophagy. When individuals encounter diverse endogenous stressors, such as denervation, aging, chronic inflammation, and exogenous factors, such as hypoxia, starvation, or minimal exercise, the muscle cell mitochondrial membrane potential diminishes while reactive oxygen species accumulate. This scenario prompts the initiation of multiple signaling pathways and activation of various non-coding RNAs, fostering an imbalance in mitochondrial dynamics and disrupting mitophagy in muscle cells. This, in turn, accelerates the process of muscle atrophy and degradation

Mitochondria, as a dynamic organelle network, can be properly adjusted according to external pressure to meet the energy needs of skeletal muscle survival and contraction. The primary function of mitochondria relies on OXPHOS within the electron transport chain (ETC) to generate adenosine triphosphate (ATP), which serves as the primary “fuel” for cellular metabolic activities. In addition, mitochondria also play a role in programmed cell death, apoptosis, calcium regulation, cell metabolism regulation, and other biological processes [16]. The health of skeletal muscle is closely related to the integrity and optimal function of the mitochondrial network [17]. The normal operation of this network depends on several key processes, including mitochondrial dynamics (mediated by mitochondrial fusion and fission), mitophagy (involving mitochondrial self-clearance and protection), mitochondrial biogenesis, mitochondrial-derived vesicles, and others [18, 19]. Mitochondrial dynamics maintains normal morphology and quantity through continuous fusion and fission, while mitophagy degrades damaged mitochondria through the autophagy pathway, ensuring the health of the mitochondrial network [20, 21]. However, when individuals are exposed to a variety of endogenous stresses (denervation, DMD gene mutation, chronic inflammation, aging, etc.) and exogenous stresses (such as hypoxia, starvation, minimal exercise, cancer cachexia, etc.), reactive oxygen species (ROS) and the mitochondrial membrane potential in skeletal muscle rapidly increase, resulting in mitochondrial functional network disorders, including imbalances in mitochondrial dynamics and mitochondrial autophagy damage. Consequently, skeletal muscle atrophy is often characterized by an accumulation of mitochondrial ROS and a decline in mitochondrial function. Excessive ROS can induce the oxidation of myofibrillar proteins, promoting their degradation. Additionally, elevated ROS levels can impede the initial stage of mRNA translation, thereby inhibiting the protein synthesis pathway [22]. Moreover, there is an increase in the expression of two skeletal muscle-specific E3 ubiquitin ligases: muscle-specific ring finger protein 1 (MuRF1) and muscle atrophy F-box protein (Atrogin-1). During this period, several atrophy pathways are triggered in skeletal muscle: (1) mitochondrial disorders can lead to decreased mitochondrial membrane potential and excessive production of ROS, which in turn activate oxidative stress, inflammation, protein synthesis, and degradation-related signaling pathways, triggering muscle atrophy programs [23, 24]. (2) Various non-coding RNAs accelerate skeletal muscle atrophy by targeting mitochondrial dynamics and mitophagy-related proteins, resulting in reduced mitochondrial numbers and dysfunction [25–28] (Fig. 1).

Therefore, maintaining the integrity of skeletal muscle mitochondria is crucial for skeletal muscle health and could be a potential therapeutic target for treating skeletal muscle atrophy. Restoring healthy mitochondrial function has been demonstrated to reduce muscle atrophy and improve health, especially by increasing mitochondrial dynamics and mitophagy. In this review, we concentrate on the current research status of mitochondrial dynamics and mitophagy in the context of skeletal muscle atrophy development and underscore the underlying mechanisms of action, suggesting a potential strategy for preventing and treating skeletal muscle atrophy by leveraging mitochondrial functions.

## Intermodulation of mitochondrial dynamics and mitophagy during skeletal muscle atrophy

Skeletal muscle mitochondria, as highly adaptable organelles, require continuous monitoring to maintain structural and functional integrity. Mitochondrial dynamics and mitophagy are key mechanisms for maintaining mitochondrial quality. Recent studies have demonstrated a close relationship between mitophagy and mitochondrial dynamics. Mitochondrial dynamics help to isolate damaged mitochondria from a healthy mitochondrial network. Moreover, mitophagy may be the result of mitochondrial dynamics, which helps to degrade isolated damaged or abnormal mitochondria. These two proteins are not completely independent of each other but have a cross-talk relationship and jointly maintain a healthy mitochondrial network. Under normal physiological circumstances, mitochondrial dynamics intricately maintain the delicate equilibrium of the mitochondrial network by orchestrating seamless interplay between fusion and fission events. This dynamic process ensures the fulfillment of the body’s energy synthesis demands [29]. However, during the process of skeletal muscle atrophy, there is an imbalance in mitochondrial network homeostasis. Activation of FoxO3 family proteins occurs via the AMPK pathway, which recruits two muscle-specific E3 ubiquitin ligases: MuRF1 and Atrogin-1 [23]. Consequently, this activation leads to the overexpression of the mitochondrial fission-related proteins dynamin 1 Like (Drp1) and fission mitochondrial 1 (Fis1), resulting in excessive mitochondrial fission [30]. Excessive mitochondrial fission resulting from imbalanced mitochondrial network homeostasis leads to a fragmented state of the mitochondrial network. In response, an adaptive mitophagy pathway is triggered, which selectively eliminates damaged mitochondria from the network [31]. Taken together, these studies indicate that Drp1-mediated mitochondrial fission is a prerequisite for triggering mitophagy. Conversely, the inhibition of mitochondrial fission also initiates the initiation of mitophagy. The protein Fis1, which is responsible for mitochondrial fission, plays a crucial role in the pivotal intersections that trigger mitophagy within the primary pathways of the stress response. The absence of Fis1 leads to mitochondrial swelling and excessive fusion, thereby triggering mitophagy in skeletal muscle. Mechanistically, Fis1 downregulation leads to the accumulation of polyubiquitinated proteins in muscle fibers, which bind to the LC3 protein through the LC3-interacting region (LIR), thereby inducing abnormal mitophagy and leading to muscle atrophy and degeneration [32, 33]. Moreover, Fis1 depletion can also induce mitophagy through the PINK1/Parkin-independent pathway. Fis1 deficiency causes abnormal accumulation of STX17 in mitochondria, exposing the N-terminus and promoting self-oligomerization, thereby triggering mitophagy [34].

Similarly, abnormalities in mitochondrial fusion also influence mitophagy. Several studies have shown that skeletal muscle atrophy in aged mice is partially caused by the downregulation of mitofusin2 (MFN2) expression [35, 36]. Loss of MFN2 inhibits the process of mitochondrial fusion, leading to an extensive amount of broken or swollen mitochondria in skeletal muscle, accompanied by a decrease in mitophagy flux. Interestingly, MFN2 deficiency can also induce ROS-dependent adaptive signaling pathways through the induction of hypoxia inducible factor 1 subunit alpha (HIF-1α) transcription factor and BCL2 interacting protein 3 (BNIP3), which can further compensate for the loss of mitophagy [35]. This compensatory mechanism aims to counterbalance the loss of mitophagy and minimize mitochondrial damage. Similarly, in the skeletal muscle of young mice, knockout of MFN2 results in impaired mitophagy and the manifestation of muscle aging-related phenotypes [36]. These findings indicate that changes in proteins related to mitochondrial dynamics can affect mitophagy through various mechanisms to maintain a healthy mitochondrial network.

**Fig. 2:**
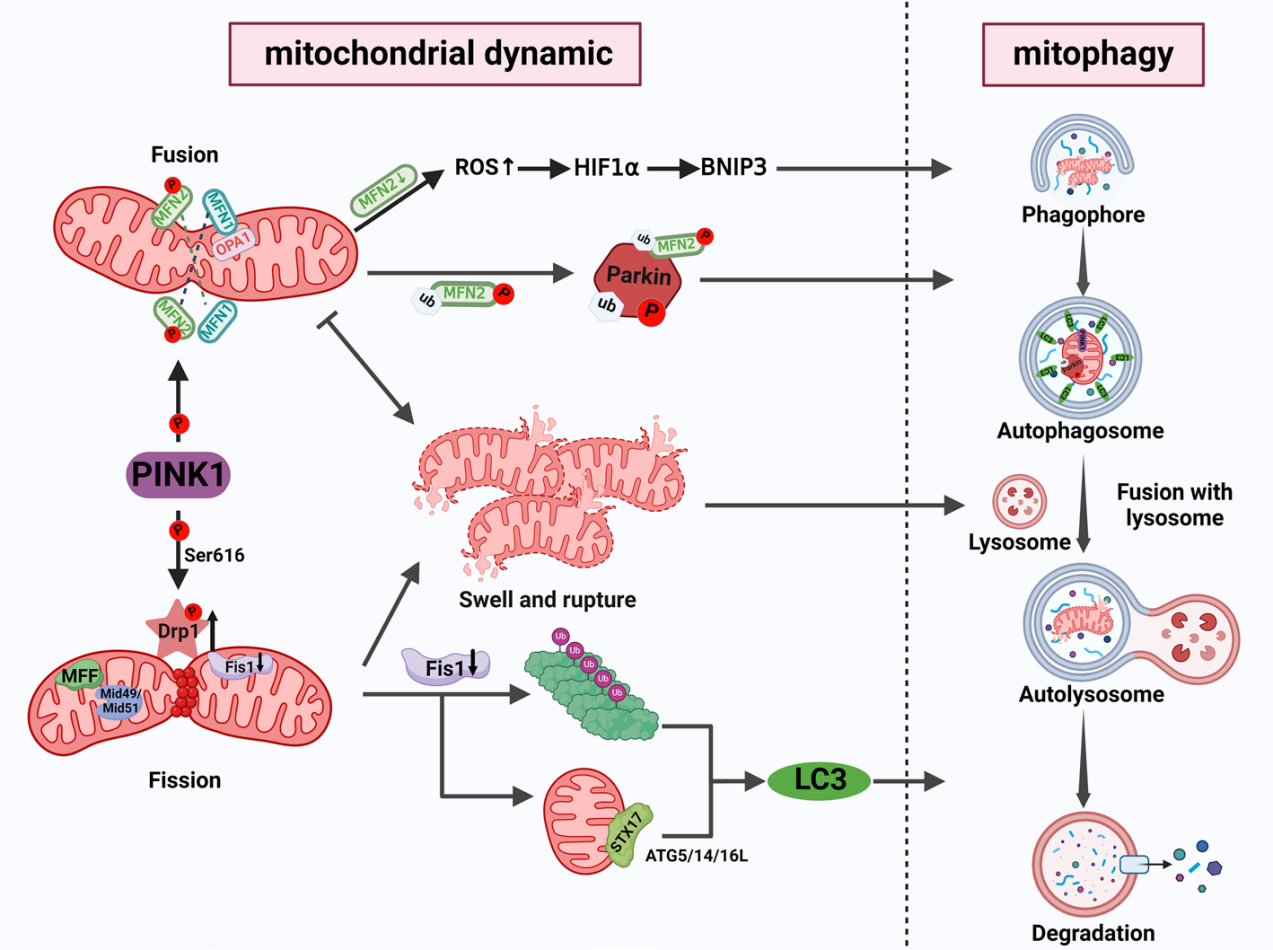
Mutual regulation of mitophagy and mitochondrial dynamics. Initially, PINK1, a pivotal protein in mitophagy, directly phosphorylates DRP1 and MFN2, facilitating the activation of both mitochondrial fission and fusion. Moreover, phosphorylated MFN2 functions as a receptor for Parkin, subsequently initiating mitophagy. Furthermore, any mitochondrial breakage or swelling arising from irregularities in mitochondrial fusion or fission prompts the activation of mitophagy, ensuring the sustenance of a healthy mitochondrial network

Impaired mitophagy also affects the occurrence of mitochondrial dynamics. In addition to their involvement in mitochondrial dynamics, proteins associated with mitochondrial dynamics, such as Drp1, MFN1, and MFN2, also serve as substrates for the PINK1–parkin pathway of mitochondrial autophagy. For instance, PTEN induced kinase 1 (PINK1) can directly phosphorylate Drp1 at Ser616 [37]. Indeed, Drp1 is a prerequisite for initiating mitochondrial fission [38]. In addition, MFN2 rather than MFN1 has been shown to be phosphorylated by PINK1 and to become a receptor for the ubiquitin ligase Parkin, which activates mitophagy through ubiquitination mediated degradation [39]. Similarly, mitophagy through the PINK1–parkin-independent pathway can also regulate mitochondrial dynamics. Under stress conditions, the mitophagy receptor FUNDC1 can dephosphorylate and dissociate OPA1 and subsequently bind to DNM1L, impacting mitochondrial fusion or fission [40]. Obviously, a distinct intermodulation exists between mitochondrial dynamics and mitophagy during skeletal muscle atrophy (Fig. 2).

## The physiological and pathological significance of mitochondrial dynamics in skeletal muscle atrophy

Mitochondrial dynamics involve the continuous fusion and fission of mitochondria and are now recognized as fundamental processes in mitochondrial metabolism and turnover [41]. It can be regulated by various cellular signaling pathways to ensure cell survival under different stressful conditions. Mitochondrial dynamics have provided numerous advantages to mitochondria, such as efficient transport, enhanced homogenization of mitochondrial populations, and effective oxidative phosphorylation. When this process is disrupted, it can trigger a series of diseases, including skeletal muscle atrophy [42]. Mitochondrial fusion typically leads to elongation of the mitochondria, resulting in an extended mitochondrial network and improved network connectivity. This elongation may contribute to an increased level of oxidative phosphorylation within the cell [29]. Furthermore, mitochondrial fusion helps to dilute the concentration of damaged mitochondria, preventing the accumulation of dysfunctional mitochondria. This process is crucial for maintaining the overall health and functionality of the organism [43]. On the other hand, mitochondrial fission plays a role in separating damaged mitochondria from the healthy mitochondrial network, allowing for their rejuvenation and increasing energy output efficiency [44]. Therefore, reasonable fusion and fission are crucial for the health of the mitochondrial network.

**Fig. 3:**
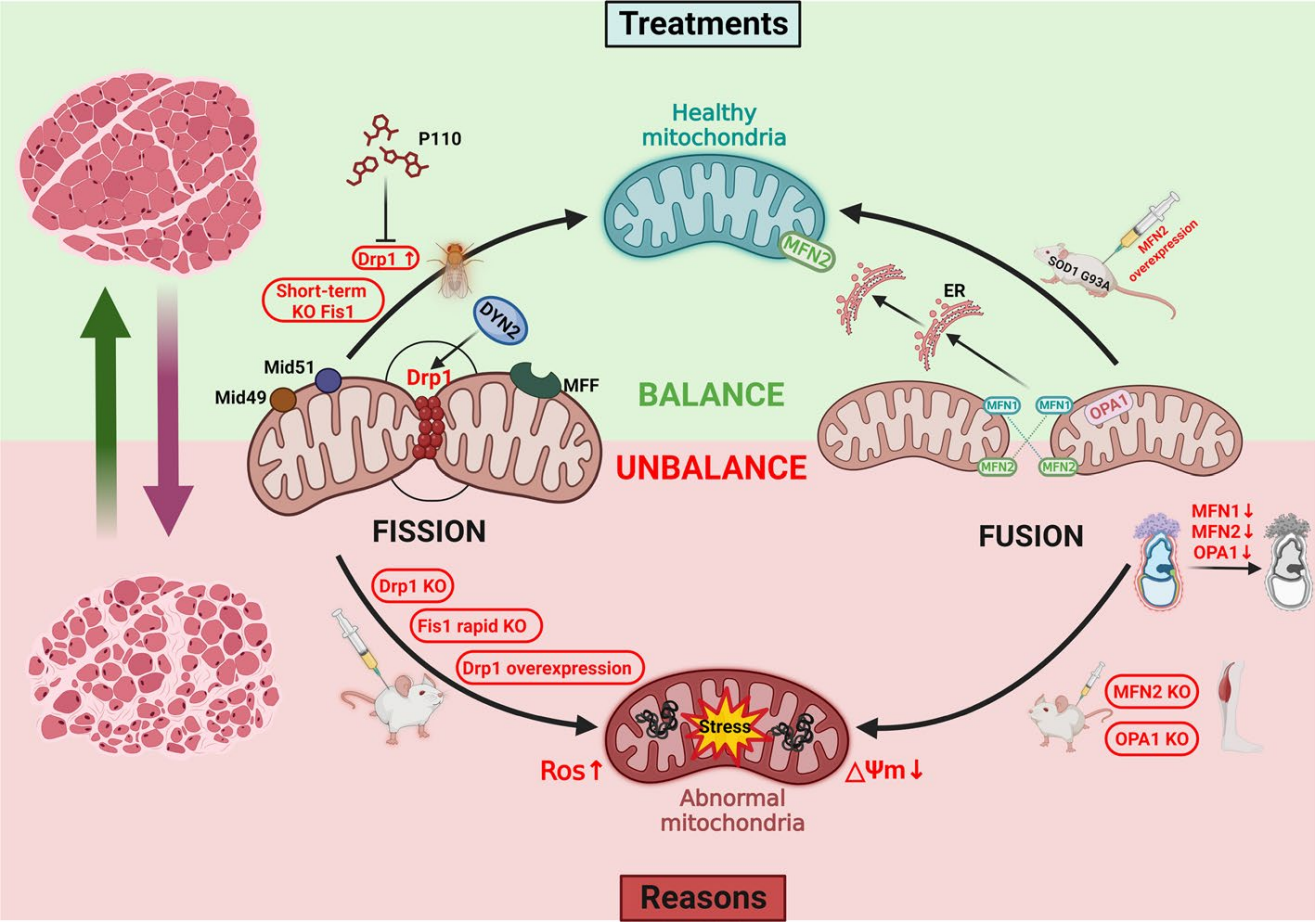
The importance of mitochondrial dynamics in maintaining skeletal muscle health. An imbalance in mitochondrial fission and fusion precipitates mitochondrial dysfunction, a primary cause of muscle atrophy and degeneration. Conversely, restoring mitochondrial dynamics through treatment in the physiological state of atrophic muscles can impede the advancement of muscle atrophy

The imbalance between mitochondrial fission and fusion leads to mitochondrial fragmentation, which is a prerequisite for mitophagy. Excessive mitochondrial fusion hinders proper mitophagy, while excessive mitochondrial fission results in an excess of independent mitochondria that are inefficient at energy production [45]. The regulation of these processes involves specific proteins, particularly members of the dynamin superfamily [46]. Key proteins involved in mitochondrial dynamics include the mitochondrial fission proteins Drp1, Fis1, MFF Mid49, and Mid51 and the mitochondrial fusion proteins MFN1/2 and optic atrophy 1 (OPA1). These proteins utilize GTP hydrolysis to drive associated proteins in the mitochondrial membrane and cytoplasm, facilitating mitochondrial fusion and fission events. Disruptions in the expression of proteins involved in mitochondrial dynamics within skeletal muscle can contribute to muscle loss under various physiological and pathological conditions, such as age-related muscle atrophy, muscle wasting associated with cancer cachexia, and sarcopenia resulting from physical inactivity [47–49]. The specific process of skeletal muscle atrophy caused by an imbalance in mitochondrial dynamics is described in the following sections (Fig. 3).

### Mitochondrial fusion dysregulation and skeletal muscle atrophy under different physiological/pathological conditions

Mitochondrial fusion is the process of fusing two adjacent mitochondrial outer membranes and inner membranes to form new mitochondria, ensuring the efficient exchange of substances within mitochondria (mitochondrial DNA, proteins, metabolites, etc.) [50]. Mitochondrial fusion consists of two crucial processes: outer membrane fusion mediated by MFN and inner membrane fusion mediated by OPA1. These processes rely on the coordination of proteins located on the outer mitochondrial membrane (OMM) and inner mitochondrial membrane (IMM). This fusion enables a greater frequency of metabolic substance exchange, the transfer of membrane potential from the inner mitochondrial membrane’s matrix side to the outer membrane, enhanced OXPHOS reactions, and reduced production of reactive oxygen species. These effects are particularly prominent in metabolically active cells, such as skeletal muscle cells, which possess a tightly interconnected mitochondrial network [51]. In addition, the excitation–contraction coupling process of skeletal muscle cells is also supported by mitochondrial fusion, and the mitochondrial fusion rate is positively correlated with the degree of skeletal muscle fiber contraction [52]. Surprisingly, the efficiency of mitochondrial fusion varies between different types of muscle fibers, with higher fusion rates observed in oxidative fibers than in glycolytic fibers [53]. The reason for this difference is that the content of mitochondria in glycolytic fibers is low. Compared with oxidative fibers, glycolytic fibers are less dependent on oxidative phosphorylation and more prone to fatigue. Consequently, the mitochondrial network is adaptively organized according to the muscle fiber type to match the metabolism and contraction characteristics of each muscle fiber type. Specifically, mitochondria in oxidative muscle fibers exhibit a lattice-like pattern, while glycolytic fibers exhibit a more fragmented mitochondrial network [53]. Interestingly, studies have shown that the types of muscle fibers involved in the process of skeletal muscle atrophy caused by different causes can often change according to different causes, including the transition from slow muscle to fast muscle and from fast muscle to slow muscle [6, 54].

In recent decades, extensive research has demonstrated the essential roles of fusion proteins, including MFN1, MFN2, and OPA1, in various cellular functions [52, 55]. The absence or reduction of these proteins has also been associated with the development of skeletal muscle atrophy under different physiological and pathological conditions [25, 56, 57]. First and foremost, abundant evidence suggests that mitochondrial fusion plays a vital biological role during early development. Notably, complete deletion of MFN1, MFN2, or OPA1 has been shown to result in embryonic lethality [58]. Second, during individual development, specific MFN2 deletion can lead to damage of neuromuscular synapses and cause skeletal muscle atrophy [59]. Conversely, it should be noted that in the study mentioned, overexpression of MFN2 specifically in neurons was found to rescue the loss of neuromuscular synapses and skeletal muscle atrophy in transgenic SOD1^G93A^ mice (a mouse model of amyotrophic lateral sclerosis) and aged mice (a mouse model of skeletal muscle natural atrophy) [59]. Similarly, a recent study demonstrated that overexpression of MFN2 in skeletal muscle of aged mice led to slight muscle hypertrophy, indicating that MFN2 overexpression may be a viable approach to mitigate age-related muscle atrophy [60]. Additionally, in humans, the loss of MFN2 and subsequent failure of mitochondrial fusion are associated with the development of Charcot–Marie–Tooth type 2A (CMT2A, a neurodegenerative disease characterized by clinical manifestations such as muscle atrophy) [61]. Although the structures and functions of MFN1 and MFN2 are similar, some studies have shown that there are some differences between the two in terms of functional regulation and disease correlation. For instance, a study conducted by Gao Song’s team revealed that MFN2 exhibits greater membrane fusion efficiency than does MFN1 in primates [61]. This disparity is attributed to the persistent dimer formation of MFN2 even after hydrolysis at the GTPase structural domain interface. Therefore, the ratio of MFN1 to MFN2 plays a significant role in modulating the mitochondrial fusion process. On the other hand, MFN2, but not MFN1, is capable of connecting the endoplasmic reticulum (ER) to mitochondria, establishing a physical foundation for important processes associated with ER–mitochondrial interactions, including calcium ion (Ca^2+^) signaling [62]. Notably, specific knockout of OPA1 in skeletal muscle can cause more severe muscle atrophy-related phenotypes than a deletion of MFN1 or MFN2 can, indicating that OPA1 can have an important impact on skeletal muscle atrophy in other biological processes other than mitochondrial fusion [56, 63]. In terms of molecular mechanisms, the loss of OPA1 in skeletal muscle can induce the accumulation of intracellular ROS and the release of mitochondrial DNA (mtDNA), which can trigger the nuclear translocation of different transcription factors, including FoxO3, NFkB, ATF4, and Nrf2. Subsequently, these transcription factors enter the nucleus and interact with skeletal muscle atrophy-related genes, FGF21 and inflammatory cytokines (e.g., IL6, IL10, TNF), resulting in skeletal muscle atrophy [56].

In summary, disruption of mitochondrial fusion in skeletal muscle leads to an imbalance between protein synthesis and degradation, resulting in accelerated skeletal muscle atrophy. These findings underscore the importance of fusion proteins in maintaining skeletal muscle health and suggest that they could be potential therapeutic targets for preventing or treating muscle atrophy.

### The function and regulation of mitochondrial fission in skeletal muscle atrophy

Mitochondrial division is a complex regulatory process that mainly depends on DRP1. This process is vital for various cellular functions, including cell fission, programmed cell death, and maintenance of healthy mitochondria [64]. The sites of mitochondrial fission occur at mitochondrial–endoplasmic reticulum (ER) contact points marked by mtDNA, and the identification of these sites relies primarily on the association between mitochondria and the ER [65]. Following identification, the DRP1 protein is recruited to the identified cleavage site, where it forms a circular protein segmentation apparatus around the mitochondria, facilitating the contraction and division of the mitochondrial membrane [66]. Mitochondrial fission is not solely driven by the DRP1 protein. In mammals, DRP1 typically collaborates with classical dynamin 2 (DYN2) to facilitate a smooth process of mitochondrial fission [67]. Although the molecular mechanism of mitochondrial fission integrity has largely unknown, studies in recent decades have shown that activation of the DRP1 protein is carried out in a variety of ways, including through interactions with receptor proteins (Fis1, MFF, MID49, and MID51) [68], posttranslational modification [69], and contact activation with various organelles [70]. These findings indicate the complexity and versatility of Drp1 regulation in orchestrating mitochondrial fission. Furthermore, a recent study employing superresolution microscopy conducted a comprehensive analysis of mitochondrial fission, resulting in the redefinition of two spatially distinct types of fission: midzone fission and peripheral fission. These terms describe whether fission occurs at central or other locations in mitochondria [64]. The study revealed that peripheral division allows damaged material to be released into smaller mitochondria for mitophagy, whereas division in the intermediate region leads to mitochondrial proliferation. This study has provided a new direction for understanding the molecular components and regulation of mitochondrial fission mechanisms.

Currently, numerous studies are investigating the crucial role of mitochondrial fission in maintaining the overall health of skeletal muscle. Both excessive and insufficient mitochondrial fission have been associated with the development of various severe human diseases, including skeletal muscle atrophy. These studies underscore the significance of maintaining a balanced mitochondrial fission process for the proper functioning of skeletal muscle and highlight the potential implications for understanding and treating related disorders. In the following section, we emphasize the progress and mechanism of mitochondrial fission in skeletal muscle atrophy through the key proteins of Drp1.

#### Function and regulation of Drp1 in skeletal muscle atrophy

Drp1 is a critical protein involved in mitochondrial fission. The targeted deletion of DRP1 in muscle has been shown to induce severe skeletal muscle atrophy by impairing protein synthesis and disrupting Ca^2+^ homeostasis between the cytoplasm and mitochondria [47, 71]. As a therapeutic target, restoring the expression of DRP1 in aging Drosophila can alleviate age-related sarcopenia by enhancing mitochondrial morphology and function in muscle tissue and promoting effective mitophagy [72]. Paradoxically, in middle-aged mice, both knockdown and overexpression of Drp1 have negative effects on mitochondrial mass, leading to several key features of muscle atrophy. These findings indicate that targeting Drp1 expression is unlikely to be a feasible approach for combating muscle atrophy [73]. Similarly, in clinical settings, extensive evidence has emerged highlighting a significant link between mitochondrial dynamics and secondary skeletal muscle atrophy in patients with amyotrophic lateral sclerosis (ALS). Dysfunction of mitochondria mediated by Drp1 overactivation is considered to be one of the key factors leading to ALS pathology [74, 75]. The contrasting findings observed in studies examining mitochondrial fission in both mammals and Drosophila add an intriguing layer of complexity to our understanding. Ultimately, these conflicting results underscore the importance of maintaining mitochondrial division within the physiological context to ensure the integrity of skeletal muscle mass and function.

#### Unveiling the role and control of Fis1 in skeletal muscle atrophy

Within yeast, Fis1 acts as the exclusive receptor for Drp1 located on the outer mitochondrial membrane, facilitating the process of mitochondrial division by increasing the cytoplasmic protein Dnm1 (Dynamin1) [76]. In mammals, Fis1 is the most studied but not the only participant in mitochondrial fission. Rather, receptors such as MFF, Mid49, and Mid51 have emerged, suggesting the responsibility of ushering Drp1 to the outer mitochondrial membrane [77, 78]. Individual aging and long-term sedentary behavior can induce a mitochondrial fission phenotype characterized by increased Fis1 expression, accompanied by muscle mass loss [79, 80]. Several human studies have demonstrated that aerobic exercise training can remodel skeletal muscle mitochondrial fission in aging and sedentary populations, promoting the development of mitochondria into a more fused tubular network structure [79, 81]. This remodeling helps maintain higher mitochondrial oxidation capacity and substrate utilization. However, it is worth noting that other forms of exercise, such as resistance exercise, may not induce similar remodeling of mitochondrial networks [82]. In the context of fasting-induced muscle atrophy, temporarily inhibiting Fis1 represents a promising strategy to impede disease progression [30]. On the other hand, acute deletion of Fis1 in skeletal muscle has detrimental effects. This action typically triggers excessive mitochondrial fusion, resulting in ultrastructural anomalies, heightened ROS accumulation within mitochondria, reduced mitochondrial respiration, and an aggregation of polyubiquitinated proteins in muscle fibers. Ultimately, this cascade leads to muscle atrophy, degeneration, diminished mobility, and a shortened lifespan [32, 33]. Therefore, the precise regulation of Fis1 is crucial for maintaining skeletal muscle health and preventing pathological conditions associated with muscle atrophy.

### Balancing mitochondrial dynamics: a feasible strategy against skeletal muscle atrophy

Abnormal mitochondrial fusion and fission are common features of both primary and secondary skeletal muscle atrophy. This not only highlights the importance of maintaining normal mitochondrial dynamics for skeletal muscle health but also suggests that the restoration of balanced mitochondrial dynamics may reverse the onset of skeletal muscle atrophy. For example, patients with ALS often exhibit excessive activation of mitochondrial fission by Drp1, resulting in massive accumulation of fragmented mitochondria, muscle atrophy and paralysis. However, studies using an exogenous inhibitor called P110, which inhibits the interaction between Drp1 and Fis1, have shown promising results. Treatment of mice with P110 significantly improved locomotion and improved the amount of healthier muscle tissue [83]. Similarly, acute deletion of OPA1 in adults leads to impaired mitochondrial fusion and increased fission, accompanied by increased FGF21 secretion in the muscle, resulting in severe skeletal muscle atrophy [84]. Mechanistically, OPA1 deficiency leads to ER stress and mitochondrial DNA release, which triggers the upregulation of the transcription factor FGF21. Moreover, ER stress triggers skeletal muscle degradation through the unfolded protein response (UPR) and the FoxO signaling pathway [56]. Studies in mice have shown that blocking excessive mitochondrial fission by downregulating the expression of Drp1 can effectively reduce the accumulation of the muscle factor FGF21 and largely alleviate skeletal muscle atrophy caused by OPA1 deficiency [85]. Obviously, it is feasible to treat skeletal muscle atrophy by targeting mitochondrial dynamics.

In addition, the expression level of the muscle factor FGF21 is usually positively correlated with unhealthy aging and muscle atrophy [86]. Transient inhibition or overexpression of FGF21 has been found to exert a health-promoting effect on skeletal muscle and enhance its adaptation to stress responses induced by perturbations in mitochondrial dynamics [87]. For example, in mouse and C2C12 cell I/RI (ischemia‒reperfusion injury) models, overexpression of FGF21 has been shown to inhibit the activation of Drp1. This inhibition of Drp1 activation by FGF21 protects mitochondria from excessive fission and contributes to the mitigation of histological damage in muscle, improvements in serum indices, and a reduction in apoptotic cell numbers in mice [87]. Furthermore, deletion of the muscle factor FGF21 has been shown to protect against starvation‐induced muscle atrophy and weakness [88]. These results indicate that in response to the detrimental effects of imbalanced mitochondrial fusion and fission on skeletal muscle, the body activates compensatory mechanisms through coordinated pathways. This compensatory response aims to restore dysregulated mitochondrial dynamics and maintain muscle health. Therefore, it is crucial to restore mitochondrial dynamics within appropriate limits in atrophic skeletal muscle to promote muscle health and counteract the progression of muscle atrophy.

## The role of mitophagy in skeletal muscle atrophy

### Overview of the mitophagy pathway in skeletal muscle

**Fig. 4:**
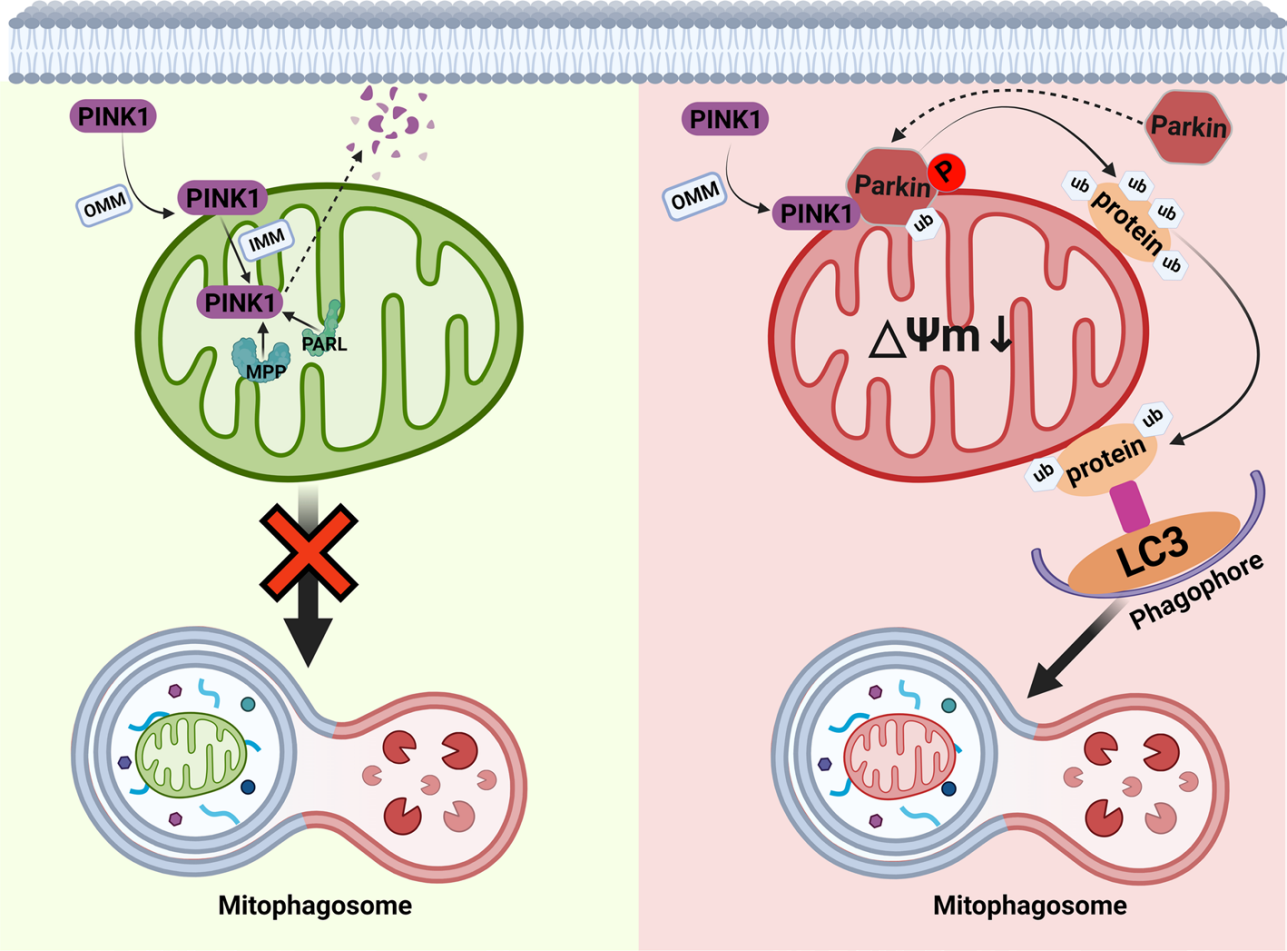
The mechanism of mitophagy under normal and abnormal physiological conditions. Under normal conditions, PINK1-mediated mitochondrial targeting and degradation inhibit parkin recruitment, thus preventing mitophagy initiation. In contrast, within damaged muscle mitochondria, PINK1 accumulation on the outer membrane triggers parkin recruitment and activation, initiating mitophagy. This cascade involves PINK1 accumulation, dimerization, and phosphorylation, which are crucial for parkin recruitment. Upon recruitment, PINK1 phosphorylates parkin at Ser65, activating it and promoting subsequent ubiquitin release

Mitophagy, a particular autophagic process mediated by the PINK1–parkin pathway that selectively eliminates extra or aberrant mitochondria, is a quality control mechanism for the removal of damaged mitochondria. PTEN-induced kinase 1 (PINK1) is a Ser/Thr kinase located in the outer mitochondrial membrane whose expression is induced by phosphatase and tens of homologs (PTEN). Parkin is a protein that is encoded by Park2 and has E3 ubiquitin protein ligase activity. Multiple studies have demonstrated that Parkin plays a protective role in maintaining skeletal muscle contraction and healthy mitochondrial function [89]. Skeletal muscle atrophy, including atrophy induced by fasting and aging, is exacerbated in the absence of Parkin or when its mitochondrial localization is reduced [90, 91]. Under normal physiological conditions, PINK1 is sequentially targeted by the mitochondrial outer membrane protein outer membrane translocase (TOM) from the mitochondrial outer membrane to the mitochondrial inner membrane and is degraded by the mitochondrial matrix processing peptidase MPP. It is then cleaved by the protease PARL in the mitochondrial inner membrane, resulting in the dissociation of PINK1 from TOM [92, 93]. Cleaved PINK1 is transferred to the cytoplasm, preventing the recruitment and activation of Parkin on the mitochondrial outer membrane (OMM), thereby inhibiting the onset of mitosis. Conversely, when mitochondria in skeletal muscle sustain damage, such as a loss of membrane potential or an excessive accumulation of ROS, the normal entry of PINK1 into the IMM is hindered. Under TOM action, PINK1 accumulates on the OMM as a dimer, where it recruits and activates Parkin and ultimately triggers mitophagy [94]. The underlying molecular mechanism involves the accumulation and dimerization of PINK1 on the OMM, which triggers autophosphorylation of Ser228 and Ser402, which is essential for the recruitment of Parkin by PINK1 [95, 96]. Once parkin is recruited to the OMM, PINK1 phosphorylates parkin at Ser65, resulting in its partial activation. Subsequently, parkin is fully activated by the addition of ubiquitin, which in turn is phosphorylated by PINK1 [97]. Mitochondrial outer membrane proteins, including VDAC1, MFN1/2, FIS1, etc., are subsequently ubiquitinated by activated parkin (parkin-interacting proteins are available in the IntAct database at https://www.ebi.ac.uk/intact). The ubiquitinated protein products, arising from parkin-mediated ubiquitination, are subsequently recruited to autophagosomes through interactions with members of the ATG8 protein family, such as LC3-II or GABARAP. These autophagosomes contain ubiquitinated cargo and then undergo fusion with lysosomes to form autophagic lysosomes. Within the autophagic lysosomes, the contents, including mitochondria exhibiting impaired ubiquitination or abnormal function, are efficiently cleared by various lysosomal enzymes [98, 99] (Fig. 4).

As mentioned above, while the PINK1–parkin pathway plays a significant role, it is not the exclusive mechanism responsible for mediating mitophagy. Other pathways are also involved in the process of mitophagy. Mitophagy can also be initiated through receptor-dependent pathways, which are characterized by the absence of parkin-mediated ubiquitin signaling [100]. For instance, various PINK1–parkin-independent receptors, including BNIP3, BNIP3L/NIX, FUNDC1, BCL2L13, PHB2, ATAD3B, and SPATA33, have been identified. These receptors are activated in response to different external stress conditions and bind to other E3 ubiquitin ligases. Then, LC3 or GABARAP proteins are recruited to connect mitochondria and autophagosomes, thereby triggering mitochondrial autophagy (these receptor proteins are listed in Table 1). This process is similar to that of the Atg32 transcription factor in yeast [101]. These proteins often possess a highly conserved LIR domain that facilitates their interaction with LC3 or GABARAP proteins [102]. Consequently, these proteins facilitate the recruitment of LC3 or GABARAP proteins, thereby establishing connections between mitochondria and autophagosomes. For example, recent studies in skeletal muscle have shown that AMBRA1 (autophagy and beclin 1 regulator 1) relies on the E3 ubiquitin ligase HUWE1 (HECT, UBA, and WWE domain containing E3 ubiquitin protein ligase 1) to induce mitophagy, and specific knockout of AMBRA1 in mouse skeletal muscle can seriously impair skeletal muscle mass and mitophagy [103, 104]. Consequently, it is imperative to expand our understanding of mitophagy beyond the PINK1–parkin pathway and consider the involvement of receptor-dependent pathway-related proteins.

**Table 1:** Summary table of proteins associated with the receptor-dependent pathway of mitophagy

Protein name	Mitochondrial localization	Inducer	References
BNIP3	OMM	Hypoxia	[105]
BNIP3L/NIX	OMM	Hypoxia	[106]
FUNDC1	OMM	Hypoxia	[107, 108]
BCL2L13	OMM	CCCP	[109, 110]
ATAD3B	OMM	Hypoxia	[111]
SPATA33	OMM	Starvation	[112]
FKBP8	OMM	Hypoxia Iron depletion	[113, 114]
AMBRA1	OMM	FCCP	[115]
PHB2	IMM	CCCP Mixture of oligomycin and antimycin A	[116, 117]
NME4	IMM	Loss of inner membrane potential	[118]
MCL1	IMM	UMI-77 Hypoxia	[119]

### Mitophagy: a potential therapeutic target for muscle atrophy

In the context of muscle physiology, mitophagy plays a crucial role in the degradation and recycling of damaged or aged mitochondria. Mitochondrial dysfunction caused by blocked mitophagy is usually considered to be one of the main factors causing skeletal muscle atrophy [120]. Given this perspective, an increasing number of studies have focused on the mechanism of mitophagy in the process of muscle atrophy and suggested that preventing and treating muscle atrophy may be useful (Table 2). These studies aimed to enhance the understanding of the intricate processes involved and provide insights into potential therapeutic strategies for the prevention and treatment of muscular dystrophy.

**Table 2:** Summary of current treatment strategies and potential interventions targeting mitochondrial dynamics and mitophagy in skeletal muscle atrophy

Classification	Therapeutic approaches	Atrophy models	Results	References
Medication	Mitochondria-targeted antioxidants	Cancer (C26 colon cancer cells) and chemotherapy-induced cachexia—chemotherapy (oxaliplatin plus 5-fluorouracil) muscle atrophy model	SS-31 prevented mitochondrial loss and abnormal autophagy/mitophagy, and muscle atrophy was alleviated	[121]
Medication	Targeting miR-142a-5p/MFN1 axis	Denervated muscle atrophy model	Restored mitophagy, apoptosis and mitochondrial function in denervated gastrocnemius muscle (note: complete recovery of muscle atrophy cannot be achieved)	[25]
Medication	Antioxidant Apigenin	Age-related muscle atrophy model	Alleviated age-related skeletal muscle atrophy by reducing oxidative stress and inhibiting overactive mitophagy	[122]
Medication	Mitophagy activator—urolithin A	Age-related muscle atrophy model	Improve muscle performance	[123]
Nutrition	Phytochemicals—tomatidine	Age-related muscle atrophy model	Increased mitophagy through the PINK1 pathway and delay muscle atrophy caused by aging	[124]
Medication	Mitophagy activators—rapamycin	Mitochondrial muscle disease model	Augmenting mitophagy is a promising therapeutic approach for muscle mitochondrial dysfunction	[125]
Medication	Antioxidants and antiinflammatory agents—isoquercitrin	Denervated muscle atrophy model	Alleviated soleus muscle atrophy and mitophagy	[126]
Medication	Anti-inflammatory agents—celecoxib	Denervated muscle atrophy model	Inhibited mitophagy and proteolysis, and ultimately alleviate denervation-induced muscle atrophy	[127]
Exercise (aerobic and resistance exercise)	Exercise training	Age-related muscle atrophy model	Exercise induced mitochondrial autophagy and enhanced mitochondrial function, and sarcopenia was alleviated	[128]
Exercise (myotube contraction)	Physical exercise (pulse simulation)	Chronic obstructive pulmonary disease (COPD) induced skeletal muscle atrophy model	Enhanced mitochondrial autophagy to prevent MuRF-1 upregulation during cigarette smoke extracts (CSEs) exposure	[129]
Exercise (high-intensity interval training)	High-intensity interval training (HIIT) and citrulline (CIT)	Aging and obesity-related muscle atrophy model	HIIT enhances markers of mitochondrial fusion and mitophagy, and the combination of HIIT with CIT results in a more pronounced increase in muscle strength	[130]
Exercise (aerobic exercise)	Aerobic exercise training (AET)	Age-related muscle atrophy model	AET increases markers of skeletal muscle size and mitochondrial biogenesis and quality control in young men (YM) and old men (OM)	[131]
Exercise (endurance exercise)	Regular endurance exercise	Age-related muscle atrophy model	Regular endurance exercise promotes mitochondrial fission, mitophagy, and oxidative phosphorylation in human skeletal muscle	[132]

#### Pharmacological modulation of mitophagy in preventing and treating skeletal muscle atrophy

Pharmacological modulation of mitophagy has emerged as a promising approach for enhancing muscle function and preventing muscle atrophy. Notably, in the context of age-related skeletal muscle atrophy, the significant accumulation of ROS in mitochondria regulates the activity of mitophagy-related proteins through oxidative modification, accompanied by oxidative damage and decreased mitochondrial membrane potential, resulting in excessive activation of mitophagy and damage to muscle tissue [133]. To address this issue, researchers have investigated pharmacological interventions aimed at rebalancing mitophagy and alleviating oxidative stress to preserve muscle integrity and function. For instance, apigenin, a natural flavonoid with potent antioxidant properties, has shown promise in alleviating age-related skeletal muscle atrophy. By reducing oxidative stress and inhibiting excessive mitophagy and apoptosis, apigenin contributes to the preservation of muscle mass and function in aging mice [122]. It is commonly observed that aging is linked to excessive mitophagy, which may result in muscle dysfunction. Theoretically, age-related skeletal muscle atrophy can be mitigated by reducing oxidative stress and inhibiting mitophagy and apoptosis [80]. However, contrasting findings have emerged from several studies, indicating that skeletal muscle mitophagy is impaired during aging. Impaired mitophagy can predictably lead to the accumulation of damaged mitochondria in vivo, disruption of the mitochondrial network, and initiation of the apoptotic program, which together lead to skeletal muscle atrophy [134, 135]. Under this physiological condition, several studies have demonstrated that urolithin A [123, 136] and tomatidine [124] can enhance muscle function by inducing mitophagy in age-related skeletal muscle atrophy. More notably, a recent study definitively highlighted that there was no discernible difference in the level of mitophagy observed in the muscles of young and aged mice [137]. These conflicting conclusions regarding mitophagy in skeletal muscle underscore the complexity of this process (Table 3). Thus, we summarize several factors contributing to the conflicting conclusions regarding mitophagy in aging skeletal muscle. First, different studies employ different subjects, experimental conditions, and technical methods, which can lead to variations in the results. Second, skeletal muscle atrophy is a prolonged process, and mitophagy may exert varying effects at distinct stages of the atrophy process. Finally, sex differences have been shown to influence mitophagy regulation [138].

**Table 3:** Summary of conflicting studies regarding changes in mitochondrial dynamics, mitophagy, and other mitochondrial functions across different muscle atrophy models

Models	Mitochondrial function	Result	References
DMD gene-induced skeletal muscle atrophy	Mitophagy	**↓**	[140–142]
DMD gene-induced skeletal muscle atrophy	Mitochondrial ROS	**↑**	[143]
DMD gene-induced skeletal muscle atrophy	Mitochondrial respiration	**↓**	[144]
Denervation-induced skeletal muscle atrophy	Mitochondrial dynamics	Fusion**↑** Fission**↓**	[145–147]
Denervation-induced skeletal muscle atrophy	Mitochondrial dynamics	Fission**↑**	[63]
Denervation-induced skeletal muscle atrophy	Mitophagy	**↑**	[148–150]
Denervation-induced skeletal muscle atrophy	Mitophagy	First **↑** Then **↓**	[151]
Denervation-induced skeletal muscle atrophy	Mitochondrial ROS	**↑**	[151]
Denervation-induced skeletal muscle atrophy	Mitochondrial respiration	**↓**	[152]
Aging-related skeletal muscle atrophy	Mitochondrial dynamics	Fusion**↑** Fission**↓**	[153, 154]
Aging-related skeletal muscle atrophy	Mitochondrial dynamics	Fission**↑** Fusion**↓**	[155]
Aging-related skeletal muscle atrophy	Mitochondrial dynamics	Fusion**↑** Fission**↑**	[80, 156]
Aging-related skeletal muscle atrophy	Mitochondrial dynamics	Fusion**↓** Fission**↓**	[27, 35, 48, 56, 157]
Aging-related skeletal muscle atrophy	Mitochondrial dynamics	Unchanged	[131, 132, 158]
Aging-related skeletal muscle atrophy	Mitophagy	**↑**	[27, 80, 91]
Aging-related skeletal muscle atrophy	Mitophagy	**↓**	[134, 135, 159]
Aging-related skeletal muscle atrophy	Mitochondrial ROS	**↑**	[160]
Aging-related skeletal muscle atrophy	Mitochondrial ROS	Unchanged	[161]
Aging-related skeletal muscle atrophy	Mitochondrial respiration	Unchanged	[158]
Starvation-induced skeletal muscle atrophy	Mitochondrial dynamics	Fusion**↓** Fission**↓**	[162, 163]
Starvation-induced skeletal muscle atrophy	Mitophagy	**↑**	[164]
Disuse-induced skeletal muscle atrophy	Mitochondrial dynamics	Fission**↓** Fusion NS	[165]
Disuse-induced skeletal muscle atrophy	Mitochondrial dynamics	Unchanged	[166, 167]
Disuse-induced skeletal muscle atrophy	Mitophagy	**↑**	[168–170]
Disuse-induced skeletal muscle atrophy	Mitophagy	NS	[165, 166, 171]
Disuse-induced skeletal muscle atrophy	Mitochondrial respiration	NS	[172]
Disuse-induced skeletal muscle atrophy	Mitochondrial respiration	**↓**	[173]
Disuse-induced skeletal muscle atrophy	Mitochondrial ROS	**↑**	[173]
Cancer cachexia-induced skeletal muscle atrophy	Mitochondrial dynamics	Fusion**↓** Fission**↓**	[163]

**↑** Indicates increase, **↓** indicates decrease, and NS indicates no significant change.

Furthermore, in cases of exogenous skeletal muscle atrophy, neurological skeletal muscle atrophy caused by nervous system injury or disease is common. Oxidative stress and inflammatory responses are typically considered early events triggering disease onset. In the treatment of neurogenic skeletal muscle atrophy, drug interventions such as antioxidants or antiinflammatories can significantly mitigate abnormal mitophagy and decelerate the progression of skeletal muscle atrophy. For instance, a range of antioxidant and antiinflammatory medications, including isoquercitrin [139], celecoxib [127], and salidroside [126], have been demonstrated to be able to impede the inflammatory response and oxidative stress in denervated skeletal muscle. Mechanistically, these medications can hinder mitophagy and proteolysis, enhance blood circulation in the targeted muscles, and ultimately ameliorate muscle atrophy resulting from denervation.

#### Stabilizing mitophagy through targeted exercise: a nondrug approach to treating skeletal muscle atrophy

In recent years, exercise therapy has also become an efficient nondrug therapy for the treatment of skeletal muscle atrophy. Numerous studies have demonstrated that targeted exercise and training can enhance mitophagy, the process of removing damaged mitochondria, thereby countering muscle atrophy caused by muscle damage or metabolic disorders. This effect is achieved through the activation of various nuclear proteins in the body, including those involved in the PINK1–parkin pathway and other related pathways. Muscle use and exercise are known to stimulate myogenesis and muscle growth. Myogenesis, driven by myoblast myogenic differentiation, is a pivotal process in skeletal muscle development and the maintenance of homeostasis. Mitophagy plays an indispensable role in facilitating this process [174]. Conversely, prolonged lack of exercise results in a higher rate of muscle protein breakdown than synthesis, leading to reduced skeletal muscle load and consequently accelerating muscle atrophy [175]. Interestingly, diverse types of exercise modulate mitophagy through distinct signaling or regulatory pathways to deter skeletal muscle atrophy. For instance, resistance exercise can prevent and mitigate skeletal muscle atrophy by triggering AMPK-mediated mitophagy and preserving the health of the mitochondrial network in skeletal muscle. Mechanistically, it downregulates the expression of E3 ubiquitin ligases, such as atrogin-1 and MuRF1, in skeletal muscle through the AMPK/FoxO3 signaling pathway and reduces skeletal muscle atrophy caused by the inhibition of protein synthesis and imbalance of ubiquitination/degradation [128, 176]. Furthermore, it has been demonstrated that aerobic/endurance exercise can mitigate skeletal muscle atrophy by promoting the mitochondrial localization of mitophagy biomarkers (including PINK1 and parkin) and the conversion of key autophagy proteins (from LC3-I to LC3-II) [128, 177]. Further evidence suggests that the enhancement of mitophagy flux in skeletal muscle induced by endurance exercise relies on parkin [178]. Different forms of exercise target the restoration of mitochondrial content and function to physiological norms by modulating mitophagy, thus mitigating the buildup of dysfunctional mitochondria and halting the advancement of skeletal muscle atrophy [176, 179].

**Fig. 5:**
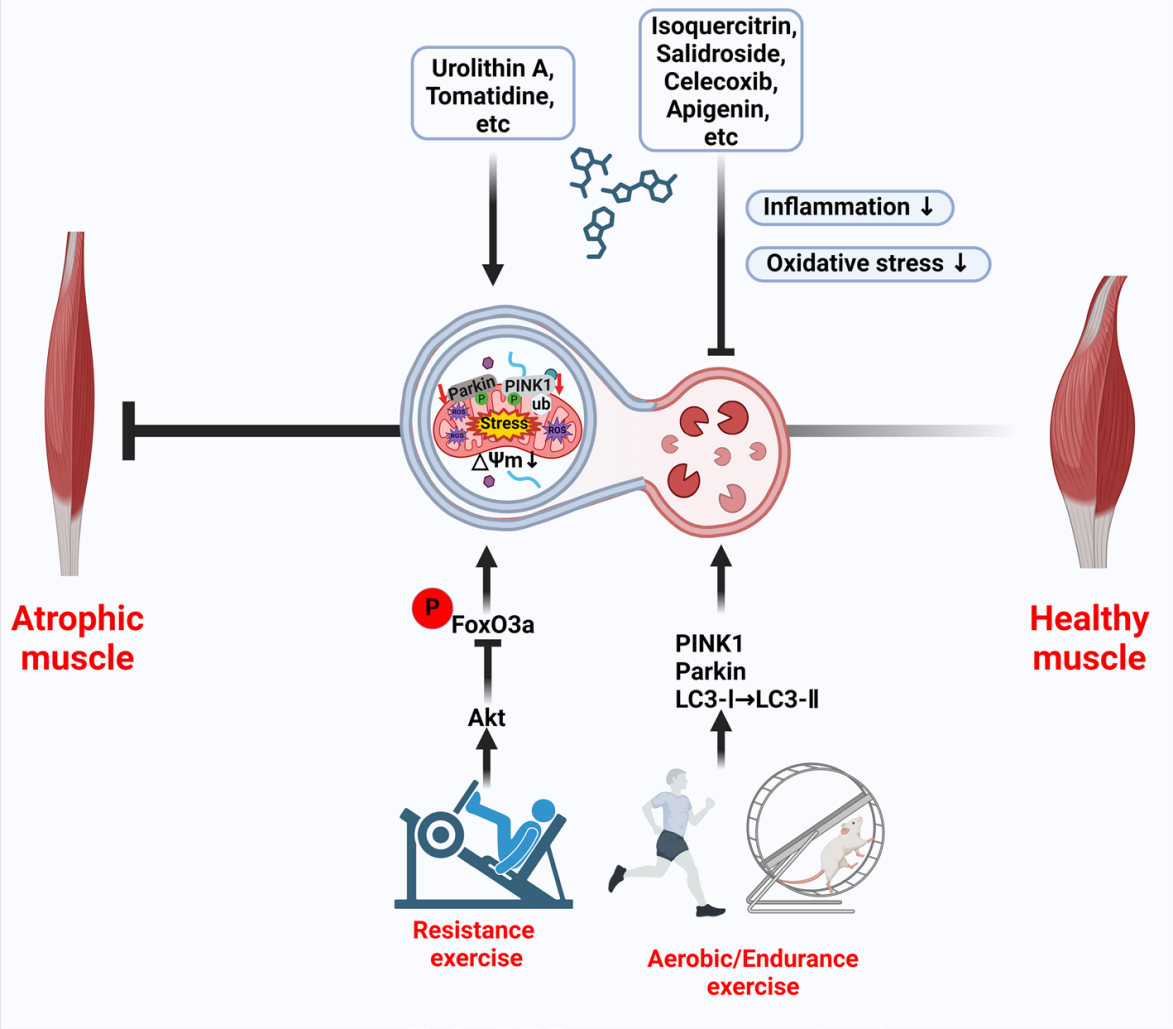
Schematic representation of potential therapeutic targets for skeletal muscle atrophy: mitophagy. Pharmacological modulation and exercise therapy are proposed as interventions to prevent and delay skeletal muscle atrophy. These drugs aim to restore physiological mitophagy levels and reduce the accumulation of damaged mitochondria in the body

Indeed, despite multiple studies examining the therapeutic effects of mitophagy on skeletal muscle atrophy induced by various causes, inconsistencies in the data persist. These conflicting results highlight the complexity of mitophagy regulation and its role in skeletal muscle atrophy, which may vary depending on the specific conditions, models, and factors under investigation. Further research is necessary to unravel the specific underlying mechanisms involved and clarify the impact of mitophagy in the context of skeletal muscle atrophy (Fig. 5).

## Conclusions

Currently, skeletal muscle atrophy is a considerable global health concern, as evidenced by notable increases in both the prevalence and mortality rates of affected individuals. Skeletal muscle is the body’s most extensive metabolic organ. The significance of mitochondrial homeostasis in supporting muscle activity and preserving muscle health is increasingly acknowledged. Key mechanisms of mitochondrial quality control, such as mitochondrial dynamics and mitophagy, play a critical role in maintaining metabolic balance and maintaining mitochondrial health in skeletal muscle. The interaction between mitochondrial dynamics and mitophagy has not been fully explored, and a comprehensive understanding of the complete mechanism governing skeletal muscle atrophy has yet to be obtained. In this review, we focus on the effects of changes in mitochondrial dynamics and mitophagy at the basic physiological level on skeletal muscle health and summarize the reasons for these conflicting data. Crucially, we discussed the potential of targeted regulation of and mitophagy as a viable strategy for treating skeletal muscle atrophy. In the future, leveraging mitochondrial dynamics and mitophagy to alleviate skeletal muscle atrophy will undoubtedly become a feasible and promising strategy.

## Data Availability

No data was used for the research described in the article.

## Abbreviations

OXPHOS: Oxidative phosphorylation
ROS: Reactive oxygen species
FoxO3: Forkhead box O3
MuRF1: Muscle-specific ring finger protein 1
Atrogin-1: Muscle atrophy F-box protein
DRP1: Dynamin 1 like
Fis1: Fission, mitochondrial 1
LIR: LC3 interaction region
MFN1: Mitofusin 1
MFN2: Mitofusin 2
HIF-1α: Hypoxia inducible factor 1 subunit alpha
BNIP3: BCL2 interacting protein 3
PINK1: PTEN induced kinase 1
OPA1: Optic atrophy 1
OMM: Outer mitochondrial membrane
IMM: Inner mitochondrial membrane
CMT2A: Charcot–Marie–Tooth type 2A
ER: Endoplasmic reticulum
Ca2+: Calcium ions
mtDNA: Mitochondrial DNA
FGF21: Fibroblast growth factor 21
MID49: Mitochondrial elongation factor 2
MID51: Mitochondrial elongation factor 1
ALS: Amyotrophic lateral sclerosis
